# Nanometer-Thick ZnO/SnO_2_ Heterostructures Grown on Alumina for H_2_S Sensing

**DOI:** 10.1021/acsanm.2c00940

**Published:** 2022-05-05

**Authors:** Mehdi Akbari-Saatlu, Marcin Procek, Claes Mattsson, Göran Thungström, Tobias Törndahl, Ben Li, Jiale Su, Wenjuan Xiong, Henry H. Radamson

**Affiliations:** † Department of Electronics Design, Mid Sweden University, Holmgatan 10, Sundsvall SE-85170, Sweden; ‡ Guangdong Greater Bay Area Institute of Integrated Circuit and System, Guangzhou 510535, China; § Key Laboratory of Microelectronic Devices & Integrated Technology, Institute of Microelectronics, Chinese Academy of Sciences, Beijing 100029, People’s Republic of China; ∥ Department of Optoelectronics, Silesian University of Technology, 2 Krzywoustego Street, Gliwice 44-100, Poland; ⊥ Department of Materials Science and Engineering, Ångström Laboratory, Uppsala University, Box 35, Uppsala SE-75103, Sweden

**Keywords:** gas sensors, ZnO/SnO_2_, heterostructures, ultrasonic spray pyrolysis, H_2_S

## Abstract

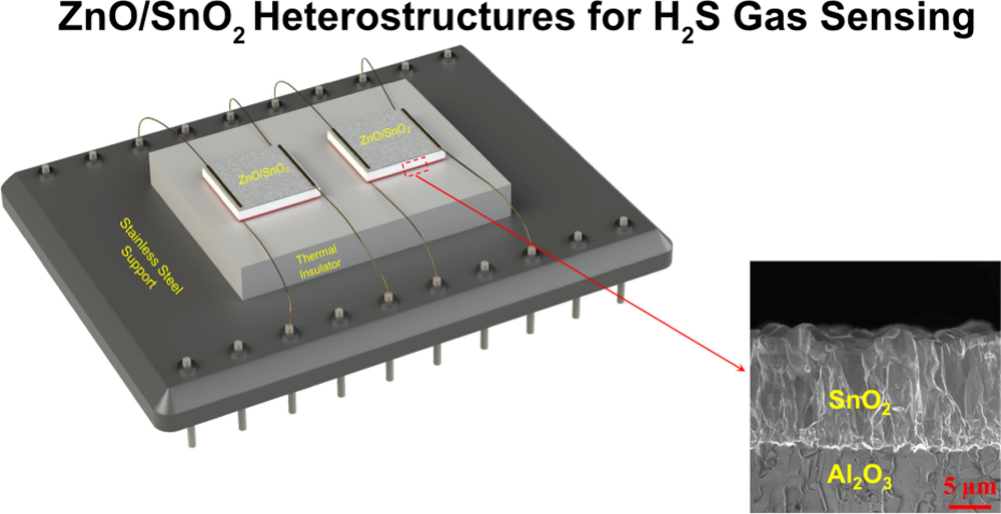

Designing heterostructure materials at the nanoscale is a well-known method to enhance gas sensing performance. In this study, a mixed solution of zinc chloride and tin (II) chloride dihydrate, dissolved in ethanol solvent, was used as the initial precursor for depositing the sensing layer on alumina substrates using the ultrasonic spray pyrolysis (USP) method. Several ZnO/SnO_2_ heterostructures were grown by applying different ratios in the initial precursors. These heterostructures were used as active materials for the sensing of H_2_S gas molecules. The results revealed that an increase in the zinc chloride in the USP precursor alters the H_2_S sensitivity of the sensor. The optimal working temperature was found to be 450 °C. The sensor, containing 5:1 (ZnCl_2_: SnCl_2_·2H_2_O) ratio in the USP precursor, demonstrates a higher response than the pure SnO_2_ (∼95 times) sample and other heterostructures. Later, the selectivity of the ZnO/SnO_2_ heterostructures toward 5 ppm NO_2_, 200 ppm methanol, and 100 ppm of CH_4_, acetone, and ethanol was also examined. The gas sensing mechanism of the ZnO/SnO_2_ was analyzed and the remarkably enhanced gas-sensing performance was mainly attributed to the heterostructure formation between ZnO and SnO_2_. The synthesized materials were also analyzed by X-ray diffraction, scanning electron microscopy, energy-dispersive X-ray, transmission electron microscopy, and X-ray photoelectron spectra to investigate the material distribution, grain size, and material quality of ZnO/SnO_2_ heterostructures.

## Introduction

It is well-known that metal oxide semiconductors are an excellent gas sensing material simply because of their high sensitivity to small concentrations of gases, long-term stability, low fabrication costs, and high potential to provide a small and low power-consuming sensor.^1−4^ In order to improve the gas sensing performance of sensors containing a single metal oxide, many research works have been performed using the homojunction between grains to study the grain size effect and morphological influence on gas sensing.^5^ However, due to the fact that the single metal oxides give an equal response to a wide range of gases, the selectivity of the sensor still remains a challenging issue.^6^ Many efforts have been focused on improving metal oxides’ selectivity by (a) doping with noble catalytic metals to promote the reaction to a specific gas, (b) surface modification, (c) using machine learning, and (d) use of multicompositional sensing films.^7,8^ In the case of multicompositional films, recent reports have indicated that by applying heterojunctions of dissimilar oxides in the form of surface decoration and/or as composites, the gas sensing behavior of resistive sensors is remarkably improved.^9^ The main point with heterostructures made by dissimilar oxides is the formation of a depletion layer in the junction. The width of this depletion layer can be changed by the exposed target gas, as a consequence of which conductivity is altered.^10^ Moreover, the oxides with different crystal structures as well as nanoparticle size may also lead to an increase in gas adsorption sites on the oxide surface and improve overall performance.^11^ Therefore, heterostructures can greatly improve the sensitivity of sensors.

For gas sensing, many studies have also demonstrated that nanomaterials, for example, Si nanowires, are also good sensors.^4,12^ They can be easily integrated with CMOS technology, especially in the form of diodes or transistors.^13,14^ However, sensors in the form of Si nanowire transistors may show low sensitivity, and the technology used for this purpose is costly and complicated for mass production.^4,15^ Therefore, resistive metal oxides are more common sensors because of their simple operation and fabrication technology.^16^

For gas sensing, there are also many applications for medical and safety purposes as well as environmental monitoring requiring robust, compact, and high-performance H_2_S gas sensors.^17,18^ It is well-known that polluted air contains dangerous gases, for example, O_3_, CO_2_, CH_a_, and CO, where H_2_S gas is corrosive and flammable with an infamous rotten egg smell.^11,19^ Exposure to even low concentrations of H_2_S over a long period of time can cause negative effects on human health such as irritation of the nose, eyes, throat, and respiratory system.^18,20^ In addition, identifying H_2_S in exhaled breath contributes to the detection of symptoms of a variety of oral diseases such as halitosis.^21^

Nanostructured metal oxides with different morphologies have been utilized for a wide range of applications.^22−25^ Among oxide materials, both SnO_2_ and ZnO can be easily grown at low temperatures. These oxide materials are commonly used in several sensors for bio and thermal applications. Because low operational temperatures are expected, SnO_2_ and ZnO recently attracted widespread attention for use as low-power gas-sensing materials.^26−28^ Among metal oxides, heterostructures such as SnO_2_/CuO, CuO/ZnO, WO_3_/NiO, ZnO/NiO, ZnO/SnO_2_, and so on. have been synthesized with different methods as an active material for H_2_S gas sensing.^6,29−33^ ZnO/SnO_2_ sensors show remarkably higher sensitivity than sensors made only from SnO_2_ or ZnO under identical experimental conditions.^6^ Therefore, this study has chosen to carry out the gas sensing performance of ZnO–SnO_2_ materials. In the case of ZnO/SnO_2_, for example, Zhu et al. reported well-designed hierarchical and highly-ordered nanobowl ZnO/SnO_2_ gas sensors which are sensitive and selective in detecting as low a concentration as 1 ppm H_2_S gas with long-term stability and repeatability.^34^ The hierarchical sensing materials were synthesized via a sequential process combining hard template processing, atomic-layer deposition, and hydrothermal processing.^34^ Furthermore, Guo et al. published a study about the hydrothermal synthesis of ZnO/SnO_2_ for H_2_S detection.^35^ The results revealed that this type of heterostructure has a better H_2_S gas response and selectivity among some interfering gases, for example, NO, SO_2_, CO, CH_4_, and C_2_H_5_OH.^35^ The most important works concerning gas sensing of ZnO/SnO_2_ heterostructure are summarized in Table 1.

**Table 1:** Comparative Results of ZnO/SnO_2_ Sensors for Gas Sensing

material	concentration (ppm)	response (*R*a/*R*g)	*T* (°C)	target gas	ref.
SnO_2_/ZnO	0.5	11.5	100	H_2_S	(6)
ZnO/SnO_2_	0.5	30	450	H_2_S	this paper
SnO_2_ promoted with ZnO	0.5	4.5	350	H_2_S	(41)
ZnO/SnO_2_ heterogeneous nanospheres	0.5	3.94	300	H_2_S	(35)
SnO_2_ promoted with ZnO	0.5	0.71	350	H_2_S	(42)
Au-doped ZnO/SnO_2_ nanofibers	1	73.3	350	H_2_S	(11)
ZnO/SnO_2_ heterostructure	1	317	350	H_2_S	(10)
SnO_2_ nanobowls branched ZnO NWs	1	6.24	250	H_2_S	(34)
CuO functionalized SnO_2_–ZnO core–shell NWs	10	1.69	RT	H_2_S	(43)
SnO_2_–ZnO core–shell NWs	25	3.08	400	ethanol	(44)
SnO_2_/ZnO hierarchical nanostructures	25	3	400	ethanol	(45)
ZnO/SnO_2_ nanofibers	50	63.3	250	H_2_S	(46)
SnO_2_–ZnO core–shell NWs	200	280	400	ethanol	(47)
SnO_2_ doped ZnO	200	40	450–500	ethanol	(48)

For metal oxides, the optimum surface temperature for proper interaction with airborne species and sensing is above 300 °C.^36^ In order to fabricate such devices, it is required that the metal oxides with a designed texture are deposited on dielectric substrates which are chemically stable at high temperatures.^26^ Alumina substrates are widely used for depositing polycrystalline functional metal oxides due to their long list of positive features, including high-temperature stabilities and excellent dielectric properties.^26^ Different techniques like molecular beam epitaxy, vapor deposition, pulsed laser ablation, sputtering, ultrasonic spray pyrolysis (USP), electrophoretic deposition, and various sol–gel methods have been utilized for depositing ZnO and SnO_2_ on alumina substrates.^37−39^ Among these methods, USP provides some degree of control on the microstructure and morphology of the grown layers with a growth rate as high as 100 nm/min.^40^ The main advantage of this method is the ability to grow continuous layers that can perfectly follow the shape of the substrate. Besides all these positive features, USP consumes less energy compared to in-vacuum deposition techniques, which makes it more interesting for gas sensing applications.^40^

To the best of our knowledge, this is the first study to demonstrate ZnO/SnO_2_ heterostructure grown on alumina substrates using USP deposition for H_2_S gas sensing purposes. In this design, the sensing layer is integrated with the heater on the rear of the sensor. The material characterization and gas sensing properties of the grown layers with different ratios of zinc chloride and tin (II) chloride dihydrate in the initial precursor are performed and discussed. The sensing layer’s thickness effect on the performance of the sensors is undeniable; however, this research mostly focuses on the heterojunction effect of the sensors. Therefore, the selectivity and gas sensing mechanism of the ZnO/SnO_2_ has been systematically investigated.

## Experimental Section

Alumina plates (3 mm × 3 mm × 0.5 mm) with 99% purity were utilized as substrates. The USP system was utilized to form both a sensing and a heating layer. The details about the USP deposition system have already been presented in our previous work.^26^ The substrate temperature of 325 °C was used for all depositions with a precursor spray rate of 4 mL/min. In the first step, the prepared chips were directly placed on the hot stage of the USP system to form microheaters. In the case of the microheater, the precursor solution was prepared by dissolving 0.2 mol/L of SnCl_2_·2H_2_O (23742.260, AnalaR NORMAPUR, VWR) in pure ethanol (99.90% v/v). The microheater was formed by USP deposition for 75 min on the rear of the sample followed by 1000 °C furnace annealing for an hour (80% N_2_ and 20% O_2_). For deposition of the sensing layer (on the front of the sample), separate precursor solutions which consist of ZnCl_2_ (29156.231, AnalaR NORMAPUR, VWR) and SnCl_2_·2H_2_O with a molarity of 0.2 mol/L were prepared using ethanol as solvent. Then different precursor solutions are prepared by mixing a proportional volume of ZnCl_2_ and SnCl_2_·2H_2_O solutions with the volume ratios as presented in Table 2. Seven samples (S0 to S6) were prepared, among which S6 contained a pure SnO_2_ layer and S0 contained the highest investigated amount of ZnCl_2_. By choosing the appropriate deposition time, the thickness of the sensing layer for all samples was kept the same (130 ± 5 nm). The details of the deposition time for the samples are also presented in Table 2.

**Table 2:** Details of USP Depositions of Sensing Layers

	S0	S1	S2	S3	S4	S5	S6
ZnCl_2_:SnCl_2_·2H_2_O precursor solution ratios (V:V)	6:1	5:1	4:2	3:3	2:4	1:5	0:6
deposition time (min)	18.00	13.12	9.00	5.70	5.78	4.80	4.00
measured Zn/Sn ratio from EDX	0.2210	0.1852	0.0998	0.0485	0.0298	0.0258	0

The scanning electron microscopy (SEM) (TESCAN MAIA3) tool was utilized to observe the morphology of the grown layers.

The X-ray diffraction (XRD) data were collected in θ–2θ and grazing incidence modes by using Cu Kα radiation in a nonfocusing geometry using a Philips X’Pert MRD tool. In order to obtain decent beam intensity and high acquisition, an X-ray mirror was applied in primary optics and a parallel-plate collimator was placed prior to the detector in the secondary optics.

To study the elemental composition and chemical state of the ZnO/SnO_2_ heterostructure materials, X-ray photoelectron spectra (XPS) (ESCALAB 250Xi, Thermo Fisher, USA) analysis was carried out in a spectrometer with a monochromatic Al-Kα X-rays source and spot size of 500 μm. All the binding energies were charge corrected by using the C 1s peak at 284.8 eV of the surface contamination carbon. The fitted peaks of O 1s XPS spectra were deconvoluted using an XPS Peak 4.1 software.

The cross-sectional view of ZnO/SnO_2_ samples was characterized by transmission electron microscopy (TEM) (Talos F200X, FEI/Thermo Fisher, USA) with an operating voltage of 200 kV. Energy-dispersive X-ray spectroscopy (EDX) was also applied in the TEM tool to determine the element content of ZnO/SnO_2_ heterostructure samples. Furthermore, nanobeam electron diffraction and selected area electron diffraction (SAD) were also employed to analyze the phase and crystalline quality in the grain.

**Figure 1:**
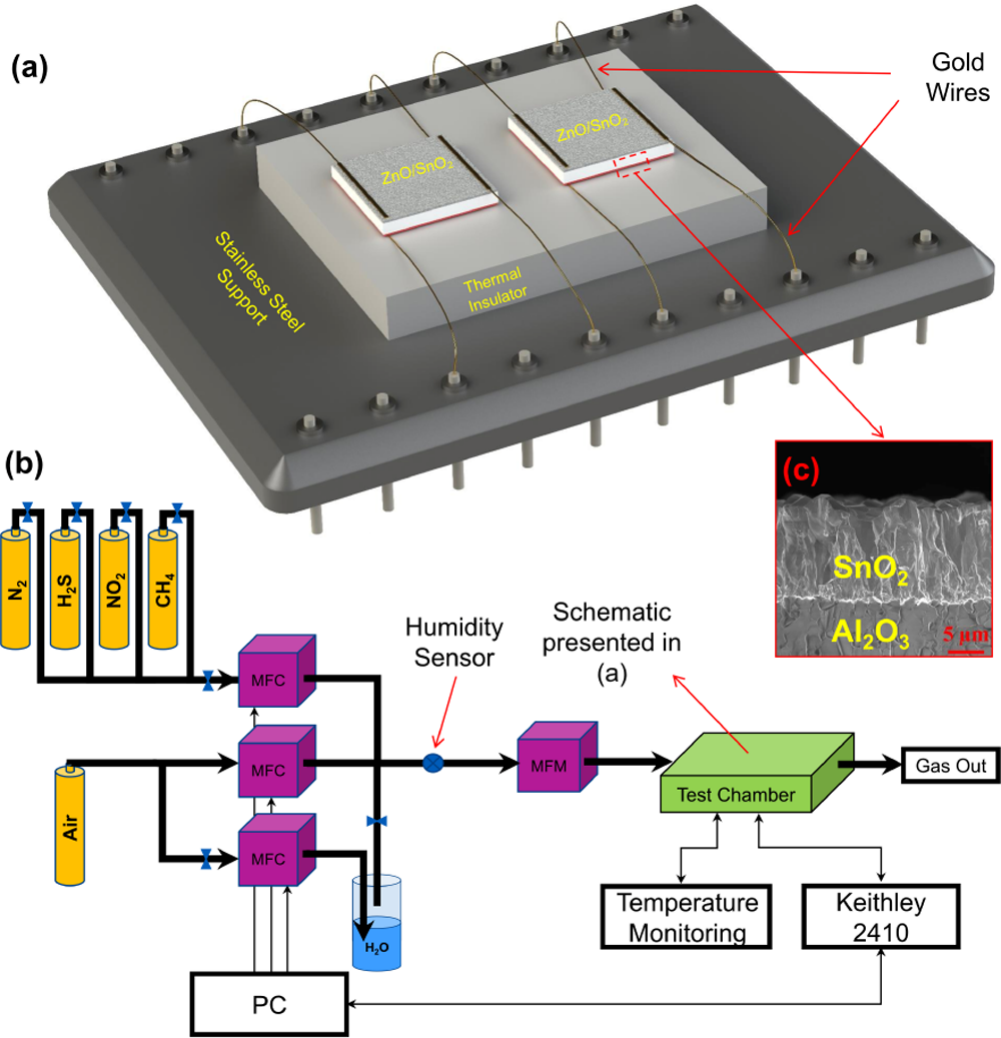
(a) Schematic image of the sample holder, (b) schematic image of the testing set-up, and (c) cross-sectional SEM micrograph of the SnO_2_ heater deposited by USP on alumina substrates.

Before gas sensing measurements, in order to stabilize the structure of the sensors, 600 °C was chosen to anneal the samples in an air atmosphere for an hour. Figure 1a displays how the prepared gas sensors were mounted on the ceramic support and electrically interconnected to a chip (25 × 35 mm^2^) with feedthroughs. Diffusion bonding of 75 μm gold wire was used to make electrical contacts within both heater and sensor structures. The sensors with their chip were mounted inside the closed chamber equipped with mass-flow controllers (see Figure 1b for more details). The cross-sectional SEM micrograph of the SnO_2_ heater deposited by the USP technique at 325 °C for 75 min is presented in Figure 1c.

Gas sensing measurements were done using a mass flowmeter and apparatus described in our previous work.^49^ The sensor temperature and electrical signal were measured by an s-type thermocouple (Pt and 90%Pt10%Rh wires with 25 μm diameter) and Keithley 2410 electrometer, respectively.

The response calculated according to
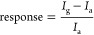
1where *I*_g_ and *I*_a_ are the sensor currents in target gas and atmospheric air, respectively.

## Results and Discussion

**Figure 2:**
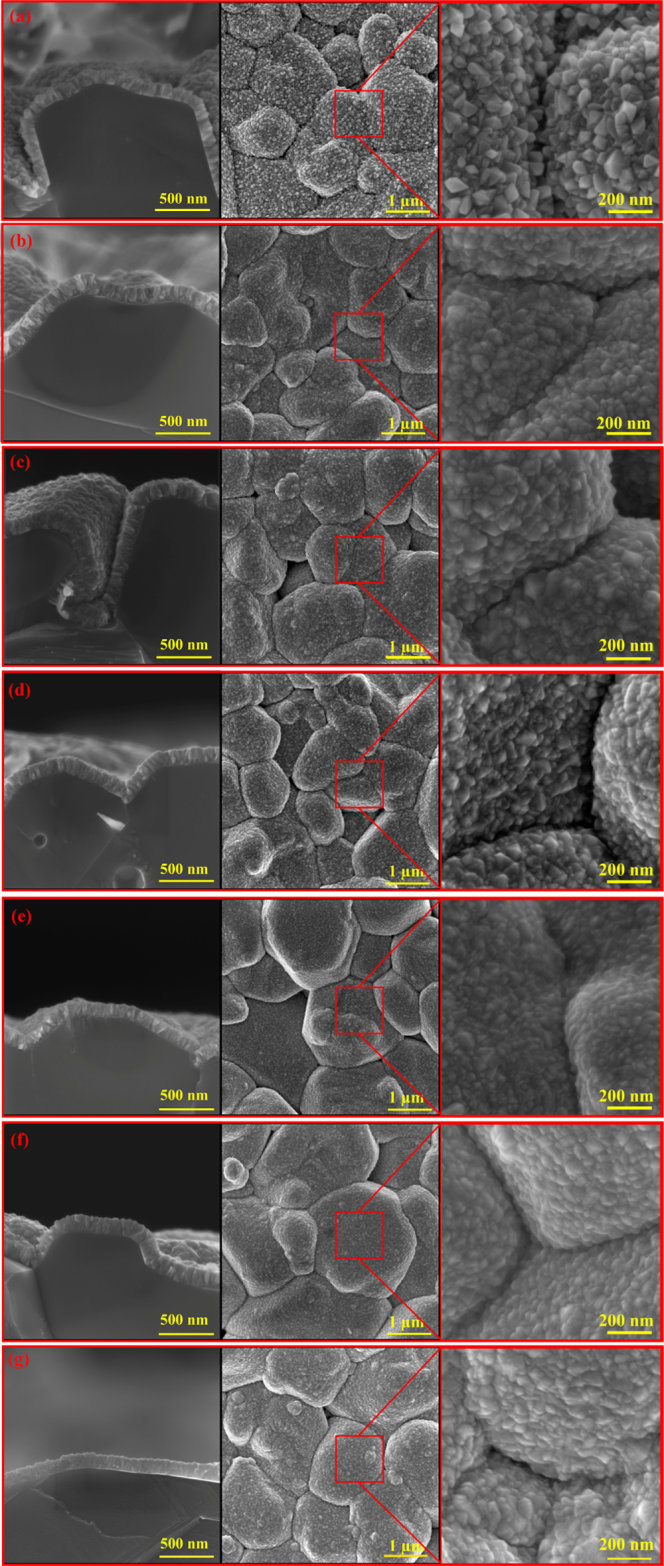
The cross-sectional and plan view SEM micrographs of the sensors prepared by USP at 325 °C on alumina substrates: (a) pure SnO_2_ (S6), (b) S5, (c) S4, (d) S3, (e) S2, (f) S1, and (g) S0.

Figure 2 displays the SEM cross-section micrographs (130 ± 5 nm thickness) of ZnO/SnO_2_ heterostructure and the corresponding top view plane. The inset images in Figure 2 at higher magnification show the morphology of the grown crystallites. According to our previous work,^26^ ZnO crystallites do not grow continuously on alumina substrates, while SnO_2_ crystallites perfectly grow on alumina substrates.^26^ This behavior also applies to the heterostructure growth of ZnO/SnO_2_. This means that for the sensors with a lower amount of SnCl_2_·2H_2_O in the initial precursor, it is necessary to increase the deposition time in order to keep the thickness the same in all sensors. Previously, the effect of thickness on the gas sensing properties of the sensors has already been investigated in detail;^50^ however, in this study, the heterojunction effects on gas sensing properties are targeted. Therefore, by changing the deposition time, the thickness of the all sensors was adjusted to be around 130 nm to obtain decent gas sensing measurements.

As observed from the SEM images in Figure 2a–g, with an increase in the ZnCl_2_ ratio in the precursor the morphology of the grown layers changes slightly. However, thanks to the benefits of USP deposition, the grown crystallites in each sensor have a uniform distribution all over the substrates. In the case of pure SnO_2_ (Figure 2a) the nanocrystallites around 57 nm were found on the surface (line averaging was utilized to determine grain size). This number decreased to 47, 50, and 50 nm for the other three sensors (S5, S4, and S3 respectively) as shown in Figure 2b–d. The smallest crystallites (around 35 and 39 nm) were found on the surface of S2 and S1, respectively. Then it increased to 49 nm for S0. This result shows that by starting with pure SnO_2_ in sample S6 and continuously increasing the ZnCl_2_ ratio, the size of the crystallites decreases (up to sample S1), and then, it increases (for sample S0). These results are in good agreement with previous reports stating that by introducing Zn atoms to the SnO_2_ initial precursor solution, the size of the grown crystallites is affected.^51^

**Figure 3:**
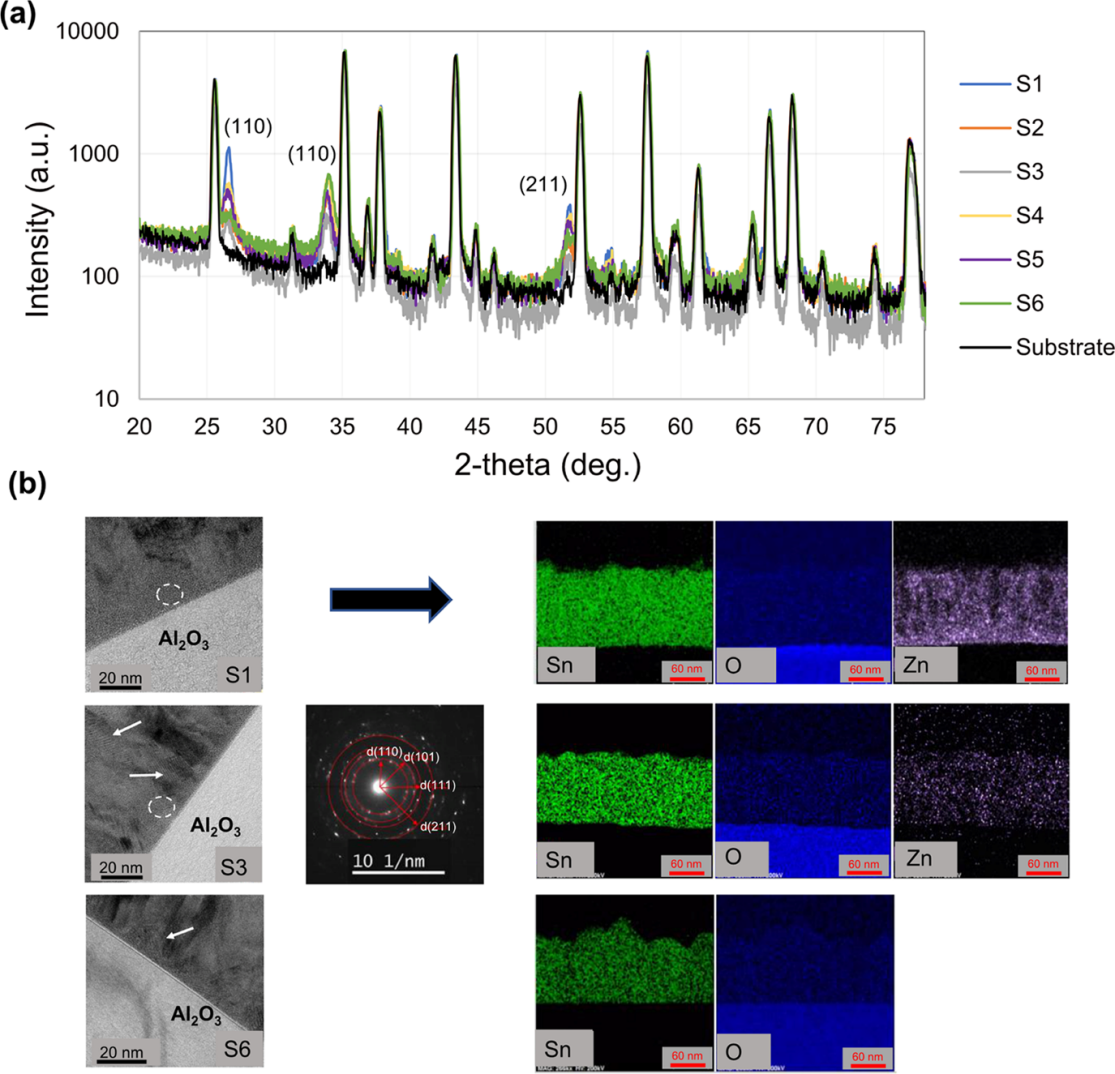
(a) θ–2θ diffractograms from sensors S1 to S6 as well as alumina substrate, and (b) TEM analysis of S1, S3, and S6 samples. The BF, SAD, and EDX images have been illustrated.

In this part, the crystal structure of the grown layers was studied by applying XRD. Figure 3a illustrates the XRD patterns of our samples. The results reveal that all samples have almost similar XRD characteristics. Apart from the substrate peaks (alumina), there are three distinguishable peaks at 26.6, 33.9, and 51.8° corresponding to the sensor layer. These peaks are indexed to the (110), (101), and (211) of the tetragonal rutile SnO_2_ phase.^51^ The absence of diffraction peaks of hexagonal ZnO can be due to the relatively small amount of ZnO grown on alumina or to the crystallites being too small to be detected by XRD. Due to the lattice mismatch of the ZnO and SnO_2,_ it is less likely to dope ZnO crystallites into the SnO_2_. Therefore, ZnO crystallites might have been grown in isolation all over the surface, forming an external heterostructure with SnO_2_ (more information will be presented later in the XPS part). In spite of the high proportion of ZnCl_2_ in the initial USP precursor, the highest intensity for (110) reflection of SnO_2_ is obtained only for S1. This may also mean that the inclusion of ZnCl_2_ in the precursor influences the morphology of the film. This may happen because of the slower deposition rate for this sample compared to the other samples. Although the thickness of the layers is the same, deposition time may affect the growth rate of some planes, such as (110). It is important to mention here that there was no detection of any crystalline ternary phases in these layers.

Further study was performed by TEM and EDX to analyze the crystallites of the sensing materials in smaller dimensions and to determine their distribution. Figure 3b shows the bright field (BF), SAD, and EDX images of S1, S3, and S6. In this figure, the SAD image of S1 is only shown because the other samples had a similar feature. The BF images reveal that the material contains mainly crystalline grains but there is a disordered area at the initial stage of the film growth (marked by dashed rings). These phases appear in SAD as concentric rings; meanwhile, there is a hollow ring, which is representative of the disordered region. The rings are identified as planes (110), (101), (111), and (211), which are also consistent with XRD results. There is also a Z-contrast in the BF images where the major distribution of SnO_2_ appear brightly and lighter elements such as ZnO are dark regions. There are stacking fault defects in the crystalline grains (marked by arrows). The EDX analysis has been focused on detecting Sn, O, and Zn elements in the samples. The presence of Zn is successively decreased from S1 to S3 until Zn vanishes completely in S6. On closer observation of sample S1, the EDX of Zn appears like bright regions containing small spots, but there are also dark regions without Zn in the layer. The Zn amount in sample S4 is obviously smaller and also nonuniform, like for sample S1.

**Figure 4:**
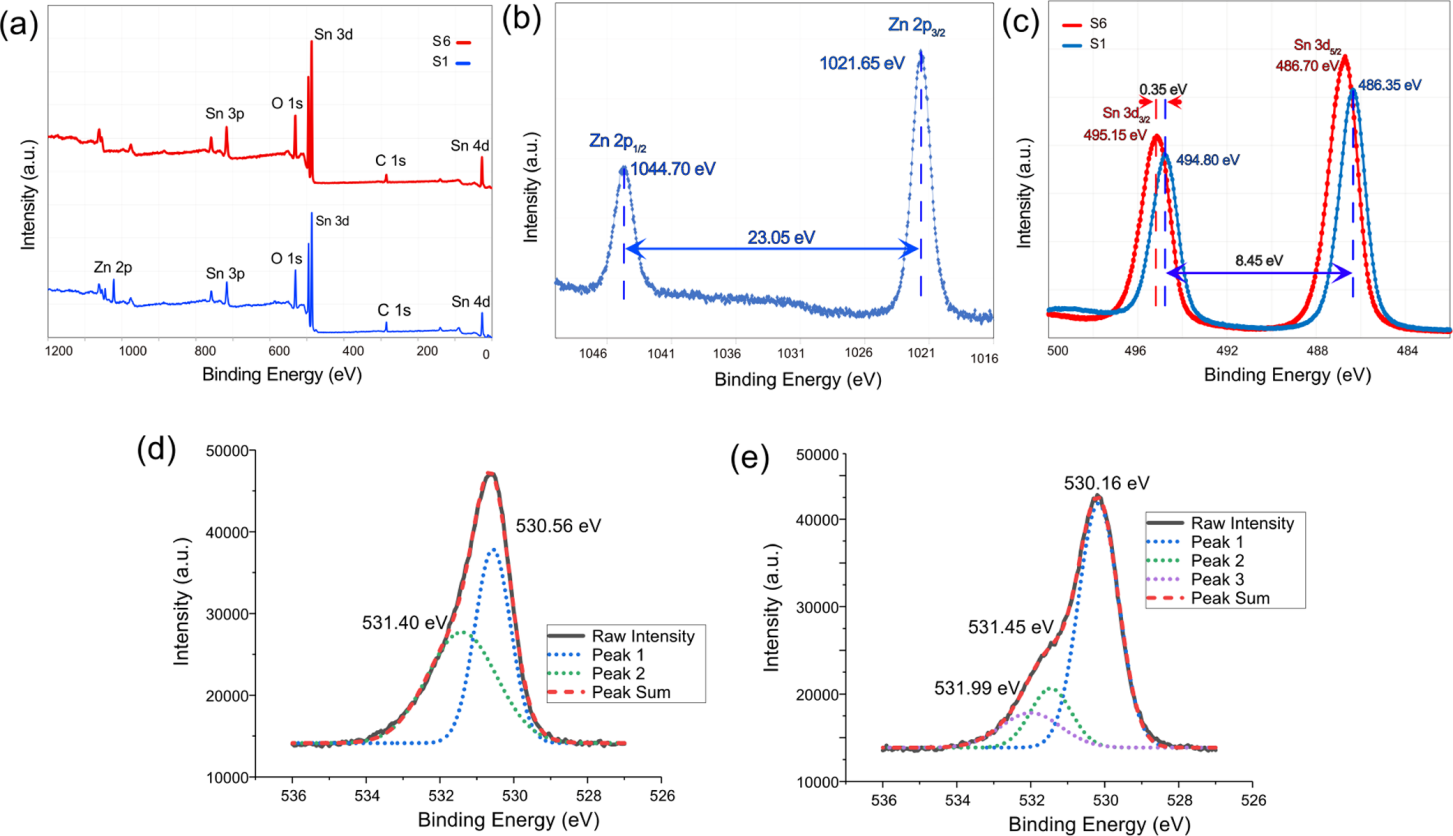
(a) XPS survey spectra of S1(pure SnO_2_) and S6 (ZnO/SnO_2_). (b) Zn 2p, (c) Sn 3d spectra for S1 and S6. (d) O 1s spectra for S6 and (e) S1.

In order to validate the chemical states and surface elemental composition, XPS measurements of S1 and S6 were carried out. As shown in Figure 4a, the peaks of Zn, Sn, and O, together with C, can be clearly observed in the survey spectrum for S1, whereas peaks of Sn, O, and C are detected for S6, and no other impurities could be observed. The C signal originates mostly from surface hydrocarbons because of the exposure of samples to air or from adventitious hydrocarbons.

As shown in Figure 4b, sample S1 has two strong peaks around 1021.65 and 1044.7 eV in the Zn 2p spectrum. These peaks correspond to Zn 2p 3/2 and Zn 2p 1/2, respectively, indicating that the Zn is in the +2 valence state. In addition, the difference in binding energies between them is 23.05 eV; hence, the substance exists in the form of ZnO.^52^ The high-resolution spectrum of Sn 3d for sample S6 shows doublet binding energies at 486.70 and 495.15 eV, corresponding to Sn 3d 3/2 and Sn 3d 5/2, respectively, as shown in Figure 4c. The energy difference between doublet binding energies is estimated at 8.45 eV, which is in excellent agreement with the reported values.^51,53^ Moreover, the Sn peaks of the S1 composite shift 0.35 eV to lower energies, indicating that the interaction and electron transformation may exist between ZnO and SnO_2_ until equilibrium is reached (the same level of Femi energy).^54,55^ The Sn 3d XPS peak positions confirm that the oxidation state of the Sn ions is +4 and the difference in binding energy between the two peaks (6.45 eV) indicates that the substance is SnO_2_, which is in good agreement with XRD analysis.

Oxygen species play an important role in the gas sensing ability of the sensors by changing the thickness of the depletion layer. Due to the asymmetric shape of the O 1s spectrum in both S1 and S6, it can be deconvoluted into different symmetric peaks to verify the status of oxygen species. For S6 (SnO_2_), according to Figure 4d, O 1s can be deconvoluted into nearly two peaks at binding energies of 530.56 and 531.4 eV. The lower binding energy corresponds to the oxygen atoms located in the lattice in the form of Sn–O. The peak at 531.4 eV could be ascribed to the oxygen vacancies in the structure of regular rutile SnO_2_. In the case of S1 (ZnO/SnO_2_) O 1s can be deconvoluted into three peaks as shown in Figure 4e. The lower binging energy at 530.16 eV is attributed to the lattice oxygens in the form of Zn–O and Sn–O. The second binding energy at 531.45 eV is ascribed to the oxygen vacancies in the ZnO/SnO_2_ structure. The highest binding energy located at 531.99 eV is related to the oxygen atoms chemisorbed at the surface of synthesized materials. This indicates that the ability of chemisorbing of oxygen on the surface of S1 (ZnO/SnO_2_) is remarkably stronger than single component SnO_2_. Therefore, the surface oxygen adsorption ability of the sensor greatly contributes to the capability of reacting with target gas molecules and it has the potential to perform well as a gas sensing material.^56^

**Figure 5:**
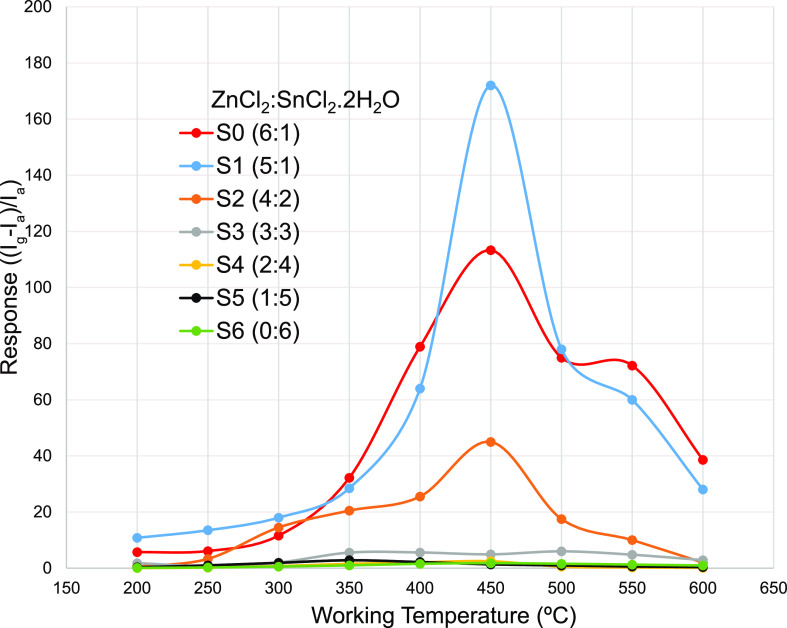
H_2_S gas sensing response of the sensors (S1 to S6) as a function of operating temperature.

The response of a gas sensor is remarkably affected by the operating temperature. Hence, the relationship between the gas sensing response and the operating temperature was first investigated in Figure 5, where all sensors are exposed to 5 ppm of H_2_S at different operating temperatures ranging from 200 to 600 °C. For sensors S3 to S6, the response is measured to be less than six according to eq 1 at all operating temperatures. For sensors S0, S1, and S2, their gas sensing responses continued to grow as the operating temperature increased from 200 to 450 °C. The maximum amount of response was obtained at 450 °C (113, 172, and 45 for S0, S1, and S2, respectively), indicating that the optimal operating temperature for this designed material combination could be chosen as 450 °C and the most sensitive sensor is S1. For this operating temperature the response and recovery time are 37, and 57 s, respectively. However, all the responses began to decrease as the operating temperature further increased above 450 °C (up to 600 °C). Basically, certain activation energy is required to have a reaction between the target gas and adsorbed oxygen species. At low operating temperatures, gas molecules are not activated enough to overcome the activation energy barrier to react with oxygen species on the surface of ZnO/SnO_2_ which leads to low response and longer response and recovery time. By increasing the temperature, the conversion of surface-adsorbed oxygen species and higher reaction activity contribute to the higher response. By increasing the temperature above the operating temperature, H_2_S gas adsorption is too difficult to be adequately compensated for the increased surface reactivity which causes a low utilization rate of the sensing material.^57^ In addition, at higher operating temperatures the response and recovery time are shorter, but the response is lower, and because of the higher operating temperature, more energy has to be consumed. In this work, sensor S1 displayed the highest response at 450 °C, which may be closely related to both the establishment of heterostructure and the effective increase in the specific surface area for oxygen adsorption.

**Figure 6:**
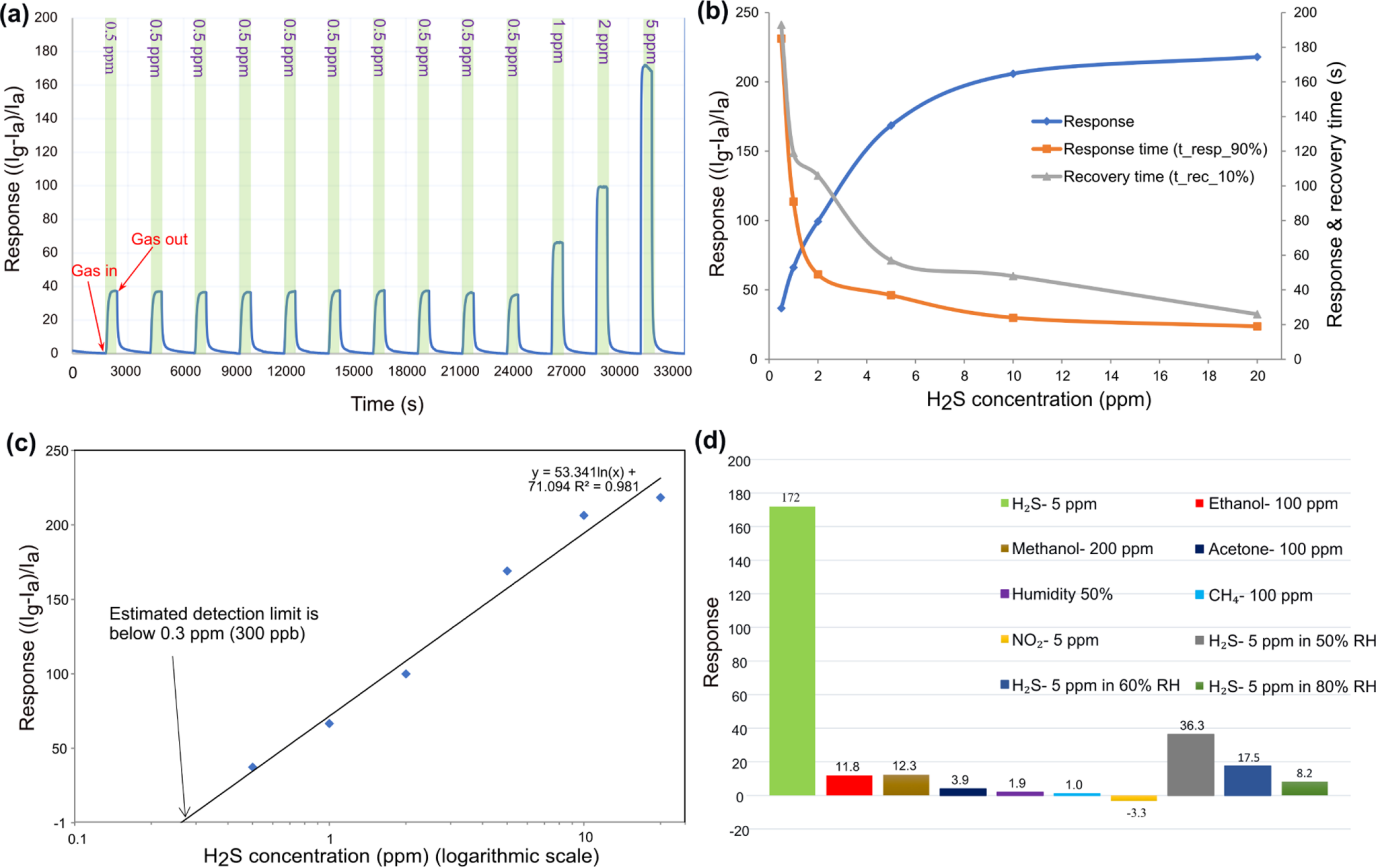
(a) Dynamic response curve of the S1 sensor for H_2_S gas with concentrations ranging from 0.5 to 5 ppm at 450 °C operating temperature. (b) Response and recovery time versus concentration, and (c) Prediction for the low detection limit of the sensor. (d) Response of S1 toward 5 ppm of H_2_S and some other interfering gases with higher concentrations.

Figure 6a shows the corresponding dynamic gas sensing response curves of the S1 (ZnO/SnO_2_) sensor at the optimal operating temperature of 450 °C, for various H_2_S concentrations ranging from 0.5 to 20 ppm. The obtained sensing response values are about 37, 66, 99, 172, 206, and 218 for 0.5, 1, 2, 5, 10, and 20 ppm H_2_S, respectively (see Figure 6b). There is a small drop in the signal for 5 ppm and this drop can be attributed to the saturation levels of different materials that we have in the heterostructure (SnO_2_ and ZnO). This saturation of the sensor starts at 5 ppm as can be observed from Figure 6b (blue line). This sensor could recover to its initial value when exposed to air again, implying satisfactory stability and reproducibility of the proposed H_2_S gas sensor. All the response times and recovery times for different concentrations of target gas are presented in Figure 6b. The repeatability of the fabricated ZnO/SnO_2_ (S1) sensor was measured to 0.5 ppm H_2_S gas at 450 °C 10 times as shown at the beginning of the dynamic response in Figure 6a (response: 36.25 ± 1.25). The measured results indicate that the response has little change. This confirms the good stability of the fabricated ZnO/SnO_2_ (S1) sensor. In addition, the long-term stability of the sensor over 30 days is presented in Figure S2. To estimate the limit of the detection (LoD) of S1 the calibration curve was prepared for the concentration range of 0.5–20 ppm (Figure 6d—blue line and Figure 6c in the logarithmic scale). The data were fitted using the logarithmic fitting (least squares method where the concentration values were logarithm), the fitted curve and its equation are presented in Figure 6c. Then the fitted line was used to extrapolate its intersection with the doubled noise level (response value of 1), this LoD is lower than 0.3 ppm of H_2_S.

The response actions of ZnO/SnO_2_ of the S1 sensor for six different gases are shown in Figure 6d. It can be seen that the highest response of the S1 sensor is observed for 5 ppm H_2_S sensing, which is almost 14 times higher than methanol, ethanol, and some other gases (with higher concentration levels, see the dynamic responses for these gases at Supporting Information, Figure S1). The reason behind the selectivity of ZnO/SnO_2_ can be attributed to the relatively small molecular size of H_2_S among most of the gases, which leads it to possess a larger adsorption capacity on the same surface adsorption area.^34^ In another work from Fu. et al. demonstrated that ZnO will react with H_2_S and transfer to ZnS leading to a larger response because the conductivity of ZnS is higher than that of ZnO.^6^ The abovementioned results demonstrate that the ZnO/SnO_2_ sensor S1 can be used for H_2_S detection. However, humidity has a negative effect on the sensor’s response. In order to have more information, sensor S1 was exposed to 5 ppm of H_2_S within relative humidity levels of 50, 60, and 80%. As presented in Figure 6d, the sensor’s responses are 36.3, 17.5, and 8.2 respectively. This means that future works are required to improve the performance of the sensor in humid conditions.

The gas-sensing mechanism of ZnO/SnO_2_ heterostructures could be described by the surface depletion and grain boundary mechanism. The gas response of the sensor in pure SnO_2_ can be originated from the homojunction barrier of the intergranular barrier in SnO_2_.^20^ In the air atmosphere, the adsorption of oxygen molecules in the form of O_2_^–^, O^–^, and O^2–^ generates an electron depletion layer at the surface, resulting in a decrease in conductivity.^4^ Upon exposure to the target gas, the reaction between H_2_S molecules and oxygen preadsorbed species on the surface of SnO_2_ can return electrons to the conduction band.^10^ This decreases the width of the depletion layer and increases conductivity. In the case of ZnO/SnO_2_ heterostructure, the sensitivity is higher than the pure SnO_2_. This behavior is mainly attributed to the heterojunctions between ZnO and SnO_2_.^35^ When dissimilar semiconducting materials, for example, SnO_2_ and ZnO, which have different Fermi levels, are brought into contact, the electrons from SnO_2_ with lower work function (Φ = 4.9 eV) transfer across the interface to ZnO with higher work function (Φ = 5.2 eV) until the Fermi levels are equilibrated.^45^ As a result, band bending and charge distribution happen at the interface of the junction. Consequently, the accumulation layer is formed on the ZnO side and a depletion layer is created on the SnO_2_ side. In order to have any conduction in the sensing material, the charge carriers have to overcome this potential barrier.^45^ Upon exposure to the air atmosphere, electrons are extracted from the conduction band of ZnO and SnO_2_, and consequently, the potential barrier between ZnO and SnO_2_ increases. When this junction is exposed to the H_2_S gas, the potential barrier decreases.^34^ Herein, this junction plays a critical role in enhancing the gas response of the sensor.

## Conclusions

Novel gas sensors made of ZnO/SnO_2_ materials on alumina substrates with high sensitivity for sensing the H_2_S gas within an operating temperature range of 200–650 °C have been presented. A heater has been also integrated into the rear of the sensor for raising the operating temperature. The performance of the sensing material was improved by optimizing the solution ratios made of zinc chloride and tin (II) chloride dihydrate. The sensor with 5:1 (ZnCl_2_: SnCl_2_·2H_2_O) ratio in the USP precursor exhibited ∼95 times better response than the pure SnO_2_ sensor toward 5 ppm H_2_S at the operating temperature of 450 °C. The enhanced gas-sensing performance was due to the heterostructure formation between ZnO and SnO_2_. The main advantage of the sensor is its inexpensive one-step USP-based fabrication process on alumina substrate and its relatively high sensitivity in the range of single ppm and sub-ppm concentrations of H_2_S without any additional functionalization. Other advantages are the integrated heating element on the rear of the sensor made with a similar process (USP) and possible miniaturization of the sensing structure for future improvement of sensor power consumption. Based on our results, the simple ZnO/SnO_2_ heterostructures made by USP are good candidates for improved selective and sensitive H_2_S detection.

## Supporting Information Available

Dynamic responses of S1 to ethanol, NO_2_, methane, methanol, and acetone and long term stability of S1 over 30 days (PDF)
