# Supplementary material for: Nanometer-Thick ZnO/SnO2 Heterostructures Grown on Alumina for H2S Sensing
Source: ACS Appl Nano Mater. 2022 May 5;5(5):6954–63. doi: 10.1021/acsanm.2c00940 (PMC9152767; doi:10.1021/acsanm.2c00940)
Supplement: Supplementary file 1 — an2c00940_si_001.pdf [file an2c00940_si_001.pdf]

Supporting Information

Nanometer-Thick ZnO/SnO_2 Heterostructures Grown on Alumina for H_2S Sensing

Mehdi Akbari-Saatlu [1*], Marcin Procek [1, 4], Claes Mattsson [1], Göran Thungström [1], Tobias Törndahl [5], Ben Li[2], Jiale Su[2, 3], Wenjuan Xiong[3*] and Henry H. Radamson [1, 2, 3*]

[1]Department of Electronics Design, Mid Sweden University, Holmgatan 10, SE-85170 Sundsvall, Sweden
[2]Guangdong Greater Bay Area Institute of Integrated Circuit and System, Guangzhou 510535, China
[3]Key Laboratory of Microelectronic Devices & Integrated Technology, Institute of Microelectronics, Chinese Academy of Sciences, Beijing 100029, People's Republic of China
[4] Department of Optoelectronics, Silesian University of Technology, 2 Krzywoustego St., 44-100 Gliwice, Poland
[5] Department of Materials Science and Engineering, Ångström Laboratory, Uppsala University, Box 35, Uppsala SE-75103, Sweden

Corresponding Authors

Mehdi Akbari-Saatlu (Email: mehdiakbari125@gmail.com, mehdi.akbarisaatlu@miun.se)
Wenjuan Xiong (Email: xiongwenjuan@ime.ac.cn)
Henry H. Radamson (Email: Henry.Radamson@miun.se)

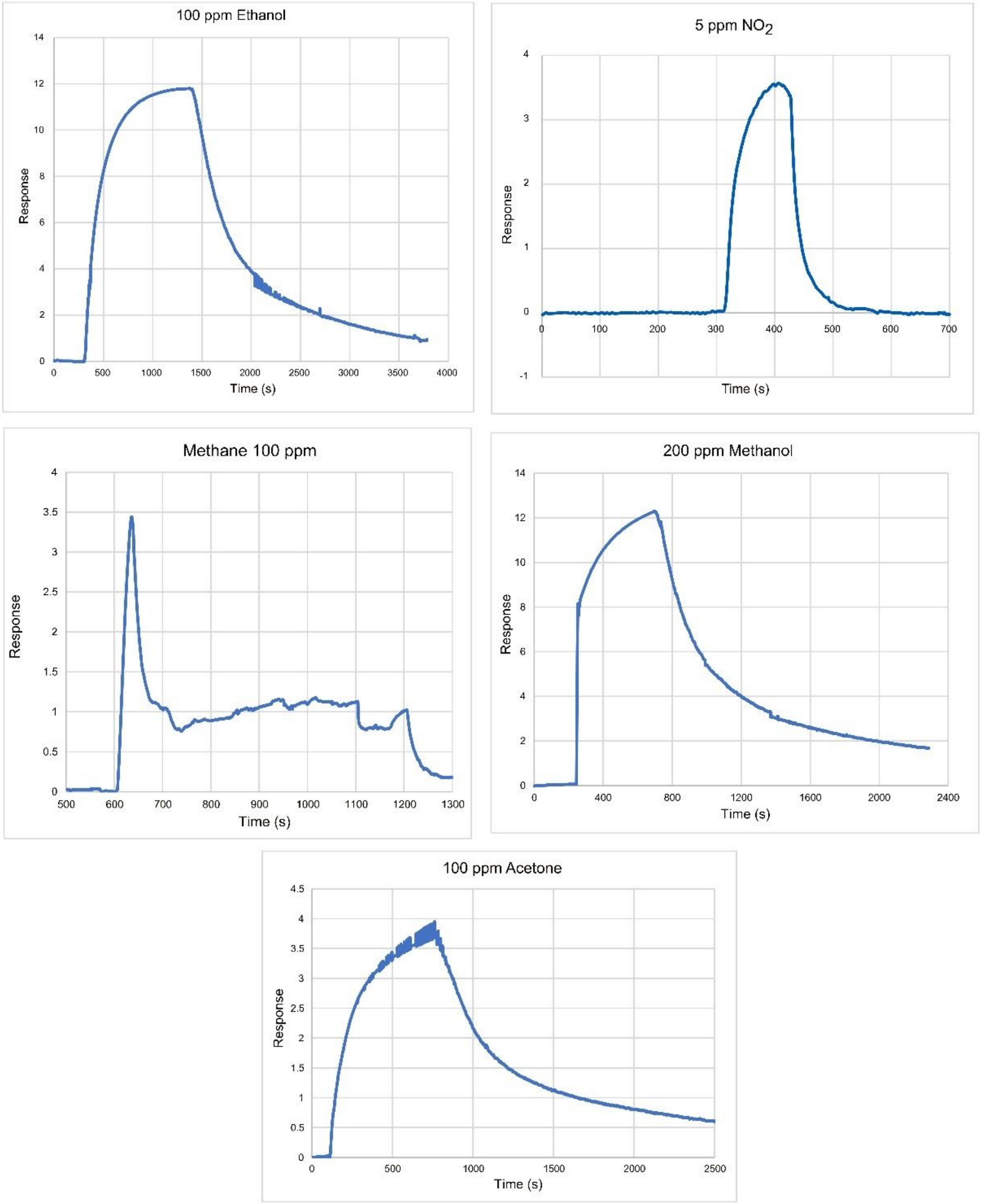

Figure S1. Dynamic responses of S1 to Ethanol, NO_2, Methane, Methanol, and Acetone at 450 °C.

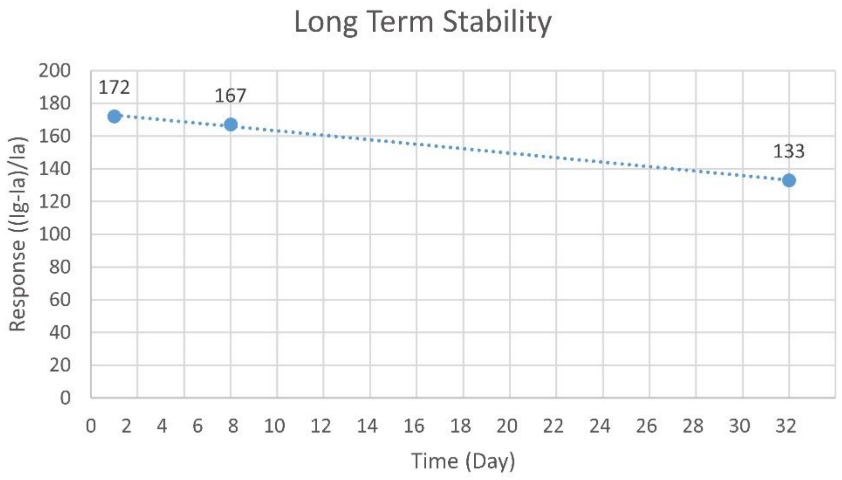

Figure S2. Long term stability of S1 over 30 days.
